# Affective temperaments and light preference

**DOI:** 10.3389/fpsyt.2025.1598849

**Published:** 2025-05-06

**Authors:** Hirofumi Hirakawa, Takeshi Terao, Kentaro Kohno, Akari Sakai, Nobuko Kawano

**Affiliations:** ^1^ Department of Neuropsychiatry, Faculty of Medicine, Oita University, Yufu, Japan; ^2^ Oita Occupational Health Management Center, Nishinihon Occupational Health Center, Oita, Japan; ^3^ Department of Psychology, Faculty of Welfare and Health Science, Oita University, Oita, Japan

**Keywords:** affective temperament, brightness preference, cyclothymic temperament, darkness preference, hyperthymic temperament, light preference

## Abstract

**Background:**

If affective temperaments are associated with light preference (brightness or darkness), such preferences may contribute to the pathophysiology of mood disorders. Moreover, light manipulation based on an individual’s temperament may offer a potential strategy for the treatment and prevention of mood disorders. This study aimed to investigate the association between affective temperaments and light preference in apparently healthy individuals.

**Methods:**

This opt-out study included data from 130 apparently healthy participants. Multiple regression analyses using the forced entry method were performed for each temperament score as the dependent variable, with age, sex, light preference (brightness or darkness), morning light exposure (yes or no), and mobile phone use before sleeping (yes or no) as independent variables.

**Results:**

Depressive, cyclothymic, and anxious temperaments were significantly associated with a preference for darkness over brightness, whereas hyperthymic temperament was significantly associated with a preference for brightness over darkness. No significant light preference was observed in individuals with an irritable temperament. The adjusted R^2^ values in the multiple regression analyses were low, indicating that the effects of light preference on temperaments were modest.

**Conclusions:**

Depressive, cyclothymic, and anxious temperaments are associated with a preference for darkness, whereas hyperthymic temperament is associated with a preference for brightness. These findings suggest that light exposure interventions tailored to temperament type may contribute to treatment and prevention of mood disorders.

## 1 Introduction

Since Kraepelin described the four basic affective dispositions—depressive, manic, cyclothymic, and irritable—as the *Grundzustaende of Manisch-Depressives Irresein* in 1913, Akiskal expanded this concept into five affective temperaments: depressive, cyclothymic, hyperthymic, irritable, and anxious (1, 2).

As for the association between affective temperaments and psychiatric symptoms, cyclothymic, depressive and anxious temperaments were significant predictors of anxiety symptoms while depressive and cyclothymic temperaments were significant predictors of depressive symptoms. Also, cyclothymic and irritable temperaments significantly predicted irritable symptoms. On the contrary, hyperthymic temperament was a significant negative predictor of anxiety and depressive symptoms (3). Regarding the association between affective temperaments and mood disorders, hyperthymic, cyclothymic, and irritable temperaments may be linked to bipolar I disorder, whereas a cyclothymic temperament may be associated with bipolar II disorder. Additionally, anxious and depressive temperaments may be associated with depression. Furthermore, cyclothymic, anxious, depressive, and irritable temperaments may be associated with emotional instability, whereas hyperthymic temperaments may be associated with emotional stability. Finally, cyclothymic, anxious, depressive, and irritable temperaments may act as risk factors for suicide, whereas a hyperthymic temperament appears to provide a protective effect against suicide (4). In addition, hyperthymic temperament may be associated with better sleep quality, whereas the cyclothymic-related temperaments may be associated with worse sleep quality (5).

Bright light therapy in the morning or exposure to ambient daylight may alleviate depression, whereas dark or virtual darkness therapy may improve mania (6). In addition, greater daytime light exposure in daily life may be associated with decreased depressive symptoms in bipolar disorder (7), whereas bedroom light exposure at night may be significantly associated with manic symptoms in bipolar disorder (8). In dementia patients exposed to morning and all-day light, night-time sleep increased significantly (mean increase: 16 minutes for morning, and 14 minutes for all-day), and morning light produced a mean phase advance of 29 minutes and evening light a mean phase delay of 15 minutes (9). In addition, illuminance may affect risk preferences, ambiguity preferences, choice consistency and dominance violations (10). Moreover, suicide rates may be negatively correlated with yearly sunshine, with an increased risk observed in regions with fewer daylight hours (11).

It is suggested that the suprachiasmatic nucleus (SCN)-independent pathway, which links intrinsically photosensitive retinal ganglion cells (ipRGCs) to the perihabenular nucleus, regulates mood through light exposure, whereas ipRGCs projecting to the SCN mediate the effects of light on circadian rhythms and learning (12). Thus, light may play an important role in the pathophysiology of mood disorders. Moreover, hyperthymic temperament has been positively associated with daytime exposure (13, 14), whereas cyclothymic temperament has been negatively associated with daytime illuminance (15).

If affective temperaments are associated with light preference (brightness or darkness), this preference may contribute to the pathophysiology of mood disorders. Moreover, mood disorders may be treated or prevented by manipulating light exposure according to an individual’s temperament. Although hyperthymic temperament has been reported to be associated with a preference for brightness (16), the light preferences of the other four temperaments remain undetermined. This study aimed to clarify light preferences for affective temperaments in apparently healthy participants.

## 2 Subjects and methods

### 2.1 Subjects

We used data from a previous study (17) on psychotherapy in apparently healthy participants. The inclusion criterion was individuals aged 20 years or older who provided written informed consent. Participants were recruited *via* electronic bulletin boards, physical bulletin boards, and flyers. Individuals with serious psychiatric disorders, as determined by the Mini-International Neuropsychiatric Interview (M.I.N.I.), were excluded. All participants included in this study were considered healthy. In this opt-out study, we analyzed data from all 130 healthy participants in the dataset, which included 108 females and 22 males, with a mean age of 49.3 years (SD = 12.1). According to M.I.N.I., there were major depression at present (N=3), major depression at past (N=2), dysthymia (N=2), hypomania at present (N=2), hypomania at past (N=4), panic disorder (N=4), agoraphobia (N=7), social phobia (N=1), generalized anxiety disorder (N=7), obsessive compulsive disorder (N=2), and alcoholic dependence (N=4), although all of them were functionally well and not serious.

This opt-out study was approved by the Ethics Committee of the Oita University Faculty of Medicine on June 12, 2023 (approval number 4).

### 2.2 Temperament assessment

Participants completed the Japanese version of the Temperament Evaluation of Memphis, Pisa, Paris, and the San Diego-auto questionnaire (TEMPS-A), a 110-item true–false questionnaire that assesses five temperament dimensions: depressive, cyclothymic, hyperthymic, irritable, and anxious (18). The TEMPS-A has been translated into Japanese, and the reliability and validity of the Japanese version have been established (19).

### 2.3 Light preference and related behaviors

A questionnaire on lifestyle habits used in previous studies (17), included a set of three questions about light such as light preference (brightness or darkness), morning light exposure (yes or no), and mobile phone use (blue light exposure) before sleeping (yes or no). These are regarded as light preference and related behaviors.

### 2.4 Data analysis

In this study, we investigated the association between affective temperaments and light preference and related behaviors using five temperament scores rated by TEMPS-A and the scores in response to three questions of light preference and related behaviors whose scores were light preference (brightness=1 or darkness=2), morning light exposure (yes=1 or no=0), and mobile phone use (blue light exposure) before sleeping (yes=1 or no=0).

For this purpose, an unpaired *t*-test was performed to compare the five temperament scores for light preference (brightness or darkness), morning light exposure (yes or no), and mobile phone use before sleeping (yes or no). Additionally, multiple regression analyses using the forced entry method were conducted for each temperament score as a dependent variable, with age, sex, light preference, morning light exposure, and mobile phone use before sleeping as independent variables.

## 3 Results

As shown in **Table 1**, participants with depressive, cyclothymic, and anxious temperaments exhibited significantly higher scores for darkness preference compared to brightness preference, whereas hyperthermic temperament showed significantly higher scores for brightness preference than darkness preference. Participants who received morning light had significantly higher hyperthermic temperament scores than those who did not. Mobile phone use before sleeping was associated with significantly higher scores for cyclothymic and irritable temperaments compared to those who did not use mobile phones.

**Table 1:** Light preference and related behavioral factors across temperaments.

Temperament	Light preference (Brightness vs. Darkness, mean ± SD)	p-value
Depressive	8.1 (3.5) vs. 10.4 (3.3)	0.004
Cyclothymic	4.4 (3.7) vs. 7.0 (5.7)	0.045
Hyperthymic	5.4 (3.5) vs. 3.7 (2.3)	0.021
Irritable	2.8 (3.1) vs. 3.7 (3.6)	0.22
Anxious	5.9 (4.9) vs. 10.2 (5.7)	0.001
Morning light exposure (Yes vs. No, mean ± SD)		
Depressive	8.4 (3.5) vs. 8.9 (3.4)	0.45
Cyclothymic	4.8 (4.3) vs. 4.8 (3.8)	0.92
Hyperthymic	5.6 (3.3) vs. 3.7 (3.3)	0.004
Irritable	2.8 (3.1) vs. 3.3 (3.3)	0.46
Anxious	6.8 (5.4) vs. 6.3 (4.9)	0.66
Mobile phone use before sleep (Yes vs. No, mean ± SD)		
Depressive	8.7 (3.4) vs. 8.4 (3.5)	0.6
Cyclothymic	5.7 (4.4) vs. 4.2 (3.9)	0.042
Hyperthymic	4.8 (2.9) vs. 5.3 (3.7)	0.43
Irritable	3.6 (3.2) vs. 2.5 (3.1)	0.045
Anxious	7.8 (5.9) vs. 6.0 (4.8)	0.06

As shown in **Table 2**, multiple regression analyses revealed that depressive, cyclothymic, and anxious temperaments were significantly associated with a preference for darkness over brightness, whereas hyperthymic temperament had a significantly positive tendency of preference for brightness over darkness. No significant preference for light was observed in irritable temperament. Additionally, hyperthymic temperament was significantly associated with morning light exposure, whereas other temperaments were not. No temperament was significantly associated with mobile phone use before sleeping. In all multiple regression analyses, the values of adjusted R^2^ were small, and variance inflation factor (VIF) values were low, ruling out multicollinearity.

**Table 2:** Multiple regression analysis for light preference and related behavioral factors across temperaments.

Depressive temperament	β	p-value	VIF
Gender (female = 1, male = 2)	–0.12	0.17	1.1
Age	–0.14	0.16	1.2
Light preference (Brightness = 1 vs. Darkness = 2)	0.24	0.009	1.1
Morning light exposure (Yes=1 vs. No=0)	–0.07	0.42	1.1
Mobile phone use before sleep (Yes=1 vs. No=0)	–0.01	0.91	1.2
Adjusted R^2^ = 0.067, p = 0.02			
Cyclothymic temperament	β	p-value	VIF
Gender (female = 1, male = 2)	0.09	0.29	1.1
Age	–0.23	0.013	1.2
Light preference (Brightness = 1 vs. Darkness = 2)	0.25	0.004	1.1
Morning light exposure (Yes=1 vs. No=0)	0.019	0.84	1.1
Mobile phone use before sleep (Yes=1 vs. No=0)	0.12	0.19	1.2
Adjusted R^2^ = 0.12, p = 0.001			
Hyperthymic temperament	β	p-value	VIF
Gender (female = 1, male = 2)	0.14	0.11	1.1
Age	0.064	0.5	1.2
Light preference (Brightness = 1 vs. Darkness = 2)	–0.15	0.084	1.1
Morning light exposure (Yes=1 vs. No=0)	0.26	0.005	1.1
Mobile phone use before sleep (Yes=1 vs. No=0)	–0.015	0.88	1.2
Adjusted R^2^ = 0.082, p = 0.009			
Irritable temperament	β	p-value	VIF
Gender (female = 1, male = 2)	0.076	0.4	1.1
Age	–0.19	0.051	1.2
Light preference (Brightness = 1 vs. Darkness = 2)	0.11	0.25	1.1
Morning light exposure (Yes=1 vs. No=0)	–0.049	0.59	1.1
Mobile phone use before sleep (Yes=1 vs. No=0)	0.12	0.21	1.2
Adjusted R^2^ = 0.048, p = 0.052			
Anxious temperament	β	p-value	VIF
Gender (female = 1, male = 2)	–0.016	0.85	1.1
Age	–0.16	0.076	1.2
Light preference (Brightness = 1 vs. Darkness = 2)	0.33	0.001	1.1
Morning light exposure (Yes=1 vs. No=0)	0.075	0.4	1.1
Mobile phone use before sleep (Yes=1 vs. No=0)	0.14	0.14	1.2
Adjusted R^2^ = 0.14, p = 0.001			

VIF, variance inflation factor.

## 4 Discussion

The findings of this study indicate a preference for darkness in depressive, cyclothymic, and anxious temperaments, whereas hyperthymic temperament was associated with a preference for brightness with significant tendency. The significant association between hyperthymic temperament and morning light exposure supports the validity of brightness preference in hyperthymic temperament, as previously reported (16). This finding is further corroborated by the study indicating that daytime illuminance, as measured by actigraphy, is positively correlated with hyperthymic temperament scores (13).

To our knowledge, this is the first study to report an association between darkness preference and cyclothymic, anxious and depressive temperaments. This may align with previous findings suggesting that these temperaments are associated with depression (4). Individuals with cyclothymic, anxiety or depressive temperaments receive less light exposure, potentially contributing to the development of depression. Encouraging greater light exposure may be beneficial in the treatment and prevention of depression particularly in individuals with cyclothymic, anxiety and depressive temperaments.

Hyperthymic temperament was significantly associated with morning light exposure, whereas other temperaments were not. There is a possibility that morning light exposure may elevate mood and maintain hyperthymic temperament, although this cannot be confirmed in this cross-sectional study.

As for light preference, people with seasonal affective disorder (SAD) preferred a more brightly lighted room than did the control group, and this light hunger (i.e., room brightness preferences) does not appear to be seasonally expressed (20). Although SAD and depressive temperament is different, light hunger in SAD is in contrast with darkness preference in depressive temperament in the present study. On the other hand, depressed patients had significantly higher photophobia scores than schizophrenics and healthy subjects (21). Although depression and depressive temperament is different, photophobia in depression is in line with darkness preference in depressive temperament in the present study.

Although “Mobile phone use before sleeping resulted in significantly higher scores for cyclothymic and irritable temperaments compared to those who did not use mobile phones.” in **Table 1**, “No temperament was significantly associated with mobile phone use before sleeping.” in **Table 2**. This is because the significant differences by unpaired t-test were adjusted by relevant factors in multiple regression analyses and thereby the significance disappeared in this case. Such changes were shown from unadjusted results in **Table 1** to adjusted ones in **Table 2** which are closer to the truth. As such, no temperament showed a significant association with mobile phone use before sleeping, suggesting no association between affective temperament and blue light exposure at night. Therefore, it is unlikely that affective temperaments influence sleep patterns through light preference.

Interestingly, individuals with irritable temperaments showed no preference for light. Whole-brain analysis revealed significant positive associations between ^18^F-FDG uptake and irritable temperament scores in the left insula and right cerebellum (Crus II, VIII, and IX), but no such association was found for depressive, cyclothymic, hyperthymic, or anxious temperament (22). This suggests that irritable temperament may differ from the other four temperaments, although this does not fully explain the lack of light preference.

As limitations, this study is cross-sectional and the causal relationship cannot be identified. Moreover, this study and analyses were not preregistered. In addition, the adjusted R^2^ values in the multiple regression analyses were small, indicating that the effects of light preference may be modest. As such, the results should be interpreted with caution.

In conclusion, depressive, cyclothymic, and anxious temperaments appear to be associated with a preference for darkness, whereas hyperthymic temperament is associated with a preference for brightness. These findings suggest that light exposure interventions tailored to temperament type may contribute to treatment and prevention of mood disorders.

## Data Availability

The original contributions presented in the study are included in the article/supplementary material. Further inquiries can be directed to the corresponding author.
